# Case Report: Delayed treatment of tuberculosis of the elbow joint

**DOI:** 10.12688/f1000research.53488.2

**Published:** 2022-05-13

**Authors:** Desdiani Desdiani, Hidayat Rizal, Anindita Basuki, Fadilah Fadilah

**Affiliations:** 1 Faculty of Medicine, Universitas Sultan Ageng Tirtayasa, Cilegon, 42434, Indonesia; 2 Department of Pulmonology and Respiratory Medicine, Bhayangkara Brimob Hospital, Depok, 16451, Indonesia; 3 Department of Orthopaedic, Bhayangkara Brimob Hospital, Depok, 16451, Indonesia; 4 Department of Radiology, Bhayangkara Brimob Hospital, Depok, 16451, Indonesia; 5 Department of Medical Chemistry, Faculty of Medicine, Universitas Indonesia, Jakarta, 10430, Indonesia

**Keywords:** Elbow, Arthritis, Tuberculosis, Delayed treatment

## Abstract

Extrapulmonary tuberculosis (TB) is known to occur in the musculoskeletal system, including the elbow joints. These cases are rarely found because the signs and symptoms are not specific to extrapulmonary TB or other diseases. We report a case of a 24-year-old male, who complained about pain in his left elbow and noticed swelling. Initially, he complained about pain all over his left arm, after several reflexology massages to alleviate his toothache. However, instead of seeking medical treatment, he visited a traditional massage therapist every week without improvement in his left arm pain including his left elbow for almost one year. Examination showed skin perforation with discharge. He also had fever during the first few days when the elbow became swollen. Weight loss and a decreased appetite were also noticed by the patient. The patient went to the orthopedic department and underwent surgery. Radiological examination indicated bone erosion on the left humerus and radius, while posteroanterior chest X-ray did not show any abnormality. Histopathological examinations from biopsy and fluid aspiration showed granulomas and datia Langhans cells. *Mycobacterium tuberculosis* was found on acid-fast bacteria smear and culture. The patient was administered multidrug tuberculosis therapy, which consisted of two months of an intensive phase and seven months of a continuation phase, in accordance with the World Health Organization’s guidelines for extrapulmonary tuberculosis treatment. He has currently undergone the continuation phase of the treatment and his condition has improved. Early detection of tuberculosis of the elbow can prevent damage to joint structure and impairment of joint function.

## Abbreviations

AFB: acid fast-bacteria

AP: anteroposterior

CKD: chronic kidney disease

CT: computed tomography

ESR: erythrocyte sedimentation rate

HIV: human immunodeficiency virus

PA: posteroanterior

TB: tuberculosis

WHO: World Health Organization

## Introduction

Extrapulmonary tuberculosis (TB) is known to occur in joints with a percentage of approximately 1-3% of all TB cases of which 2-5% are rare cases that occur in the elbow joints. ^1^ TB is an endemic disease with the total number of cases approximating 845,000 in Indonesia. ^2^ Males and females have identical rates of infection with Mycobacterium tuberculosis until adolescence, following which males have a greater rate. For all ages, male rates became higher than female rates. The average age of tuberculous vertebral osteomyelitis patients is 45–60 years old. Nonetheless, some research show a bimodal age distribution, with two peaks, one between 20 and 30 years old, linked to immigration and/or HIV infection almost 60% of cases in one study, and the other between 60 and 70 years old. ^3^ Elbow dysfunction is the result of the progressive of erosion and destruction of bone and joint, therefore early diagnosis and treatment are needed to prevent this outcome. Diagnosis is quite challenging and often late due to non-specific symptoms. ^4^ Joint TB is rarely detected because joint pain is not commonly considered to be a symptom of joint TB, especially if there are no respiratory complaints. Thus, diagnosis and treatment are often delayed. Here, we report a rare case of a patient with TB of the elbow joint, who received delayed treatment because he chose to undergo traditional treatment with massage therapy.

## Case report

A 24-year-old Indonesian male who worked in an internet rental shop came to the orthopedic department of Bhayangkara Brimob hospital (Depok, Indonesia) with left arm pain and left elbow joint swelling. Physical examination revealed skin perforation with yellowish discharge on the left elbow. The patient experienced fever on the first few days as the left elbow became swollen, weight loss, and a decreased appetite, but no respiratory complaints.

Chronologically, one year prior to coming to the hospital, the patient noticed another pain in his left arm both in the upper and lower arm. He then chose to undergo regular traditional massage therapy every week for almost one year instead of seeking for medical treatments. At the first hospital visit, the elbow pain had gotten more severe and became swollen. Within a month, discharge emerged from a small skin perforation located on the inner side of the left elbow. The patient finally went to the orthopedic department and underwent surgery. The patient had a history of undergoing reflexology massages on between the fingers of his left hand to alleviate his toothache.

**Figure 1.**
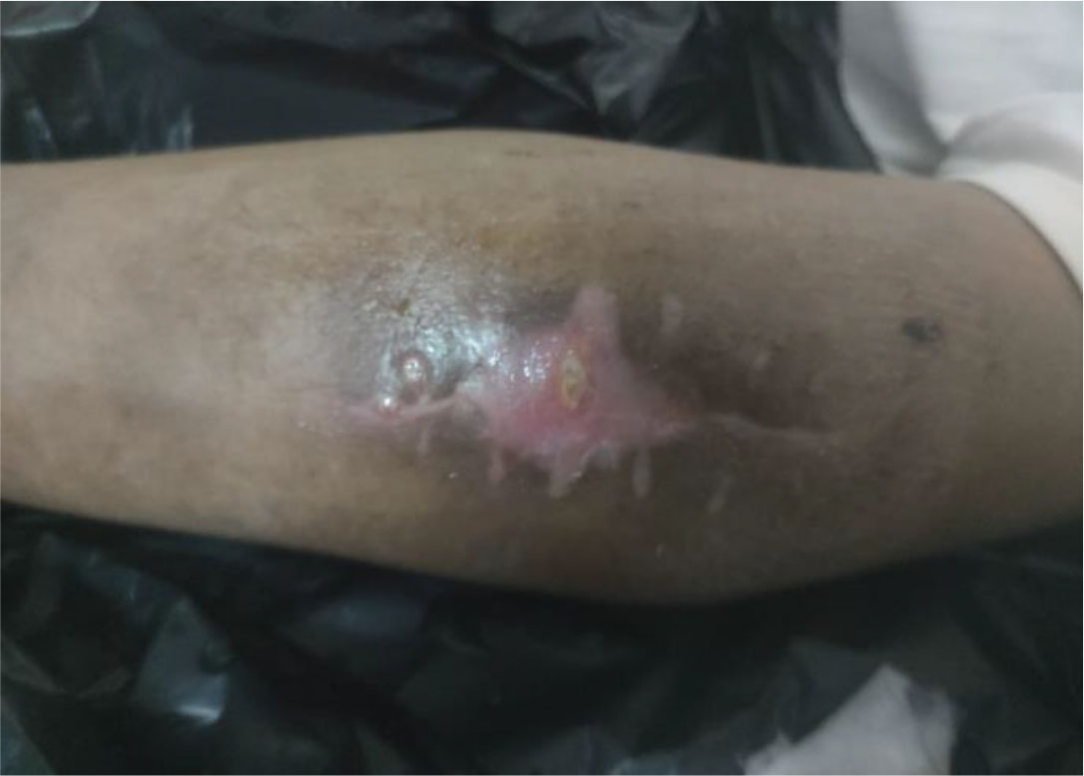
Left elbow joint appeared swollen, discharge was exuding from the perforated skin (photo after surgery).

Upon physical examination, the left elbow joint appeared swollen and discharge was exuding from the perforated skin, as depicted in Figure 1. The patient could not lift his left arm because it was be painful. Flexion and extension were also difficult due to the severity of the pain. The patient’s social environment has a culture of seek help from local traditional massage therapist who is known to be uncertified for various health problems, and instead of recovering, the patient showed symptoms that are worsening.

**Figure 2.**
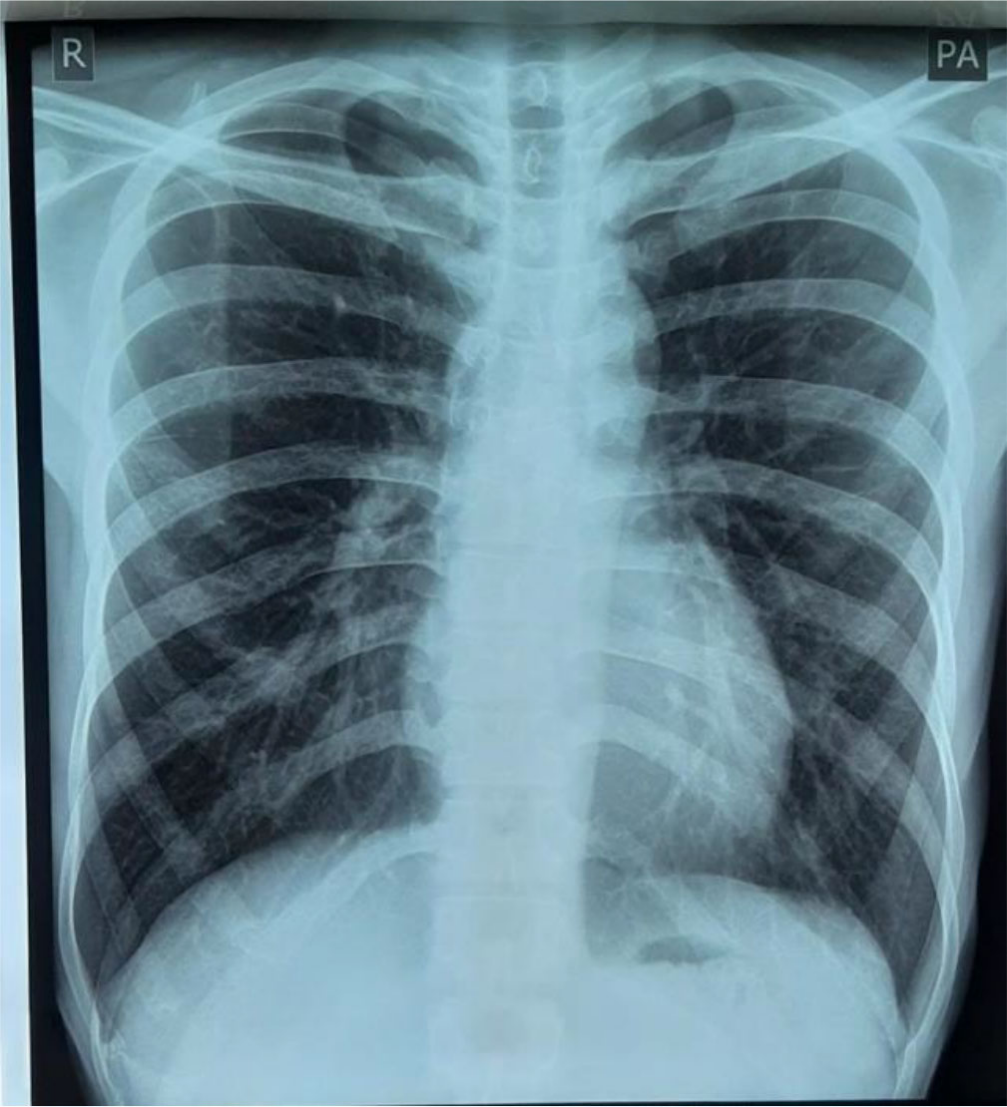
Radiological examination by posteroanterior chest X-ray did not show any abnormality.

**Figure 3.**
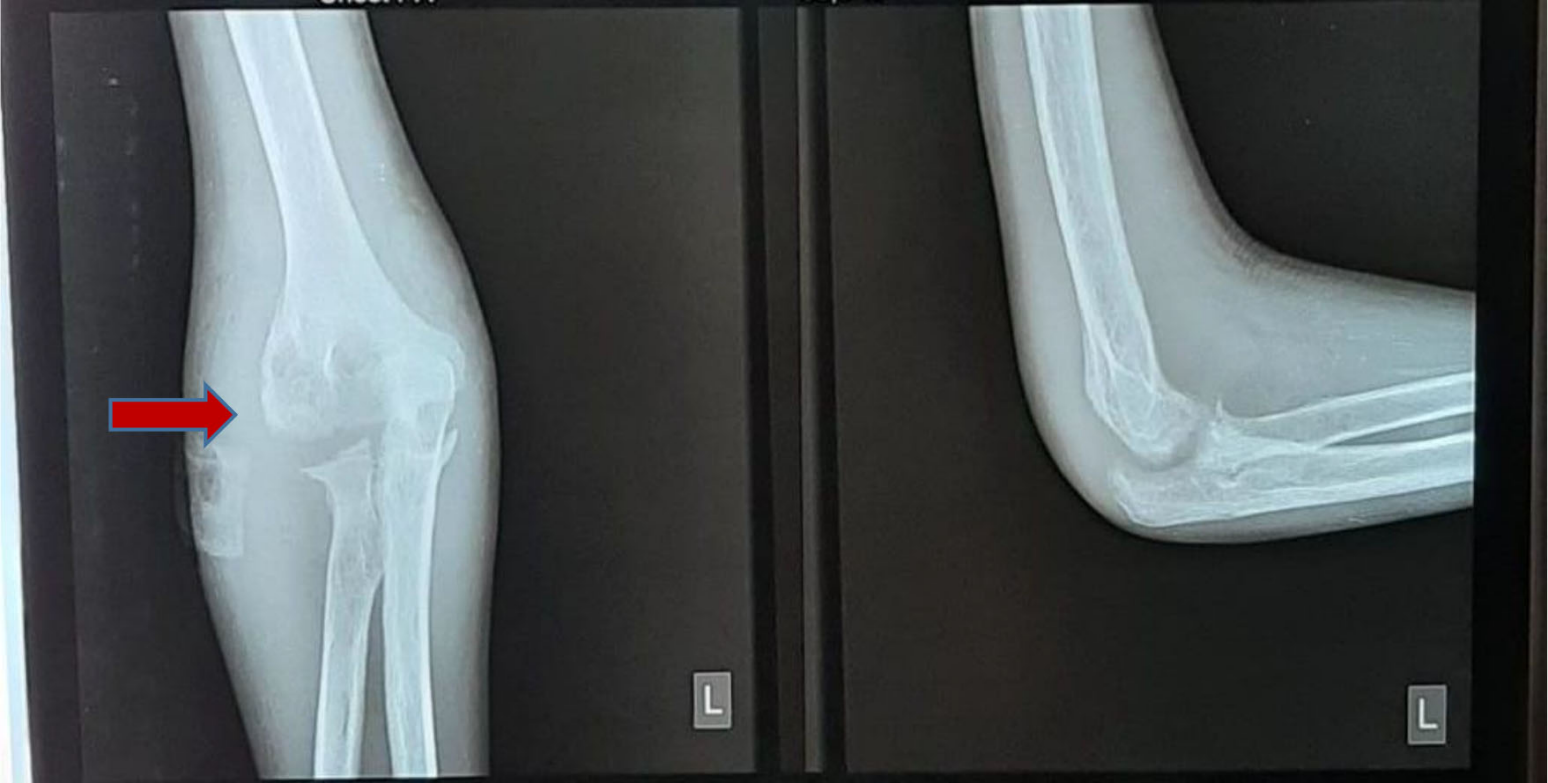
Anteroposterior and lateral projection of the left elbow joint radiographs showed erosion of the distal cortex of the humerus and radial bone, destruction of the distal cortex, and swelling of the soft tissue of the left elbow area (February 2021).

Laboratory examination revealed a leukocyte count of 15,000 (normal range: 5000-10,000 cells/μl), erythrocyte sedimentation rate (ESR) of 40 mm/hour (normal range: 0-10 mm/hour), eosinophils 9% (normal range: 1-3%), and monocytes 10% (normal range: 2-6%). Radiological examination by posteroanterior (PA) chest X-ray did not show any abnormality ( Figure 2), anteroposterior (AP) and lateral projection of the left elbow joint radiographs showed erosion of the distal cortex of the humerus and radial bone, destruction of the distal cortex, and swelling of the soft tissue of the left elbow area ( Figure 3).

**Figure 4.**
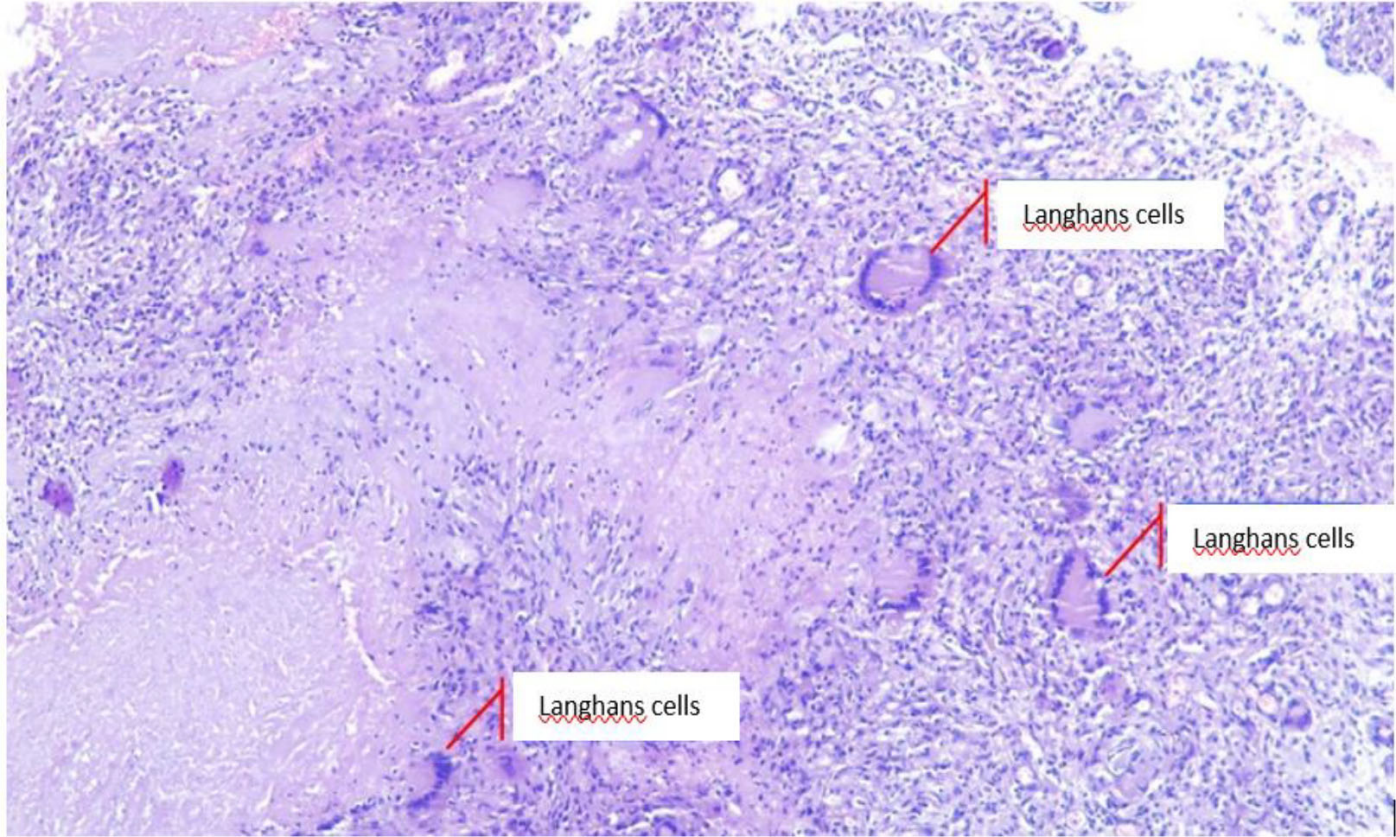
Granulomatous inflammation, swollen connective tissues containing epithelioid tubercle nests with necrotization, and datia Langhans cells.

The patient was suspected with TB of the elbow joint. He then underwent left elbow arthrotomy and synovial fluid aspiration. The surgery was performed with the patient supine under general anesthesia. Incisions were made layer by layer on the posterior region of *cubiti sinistra.* White granulation tissue and thick yellow intra- and extra-articular pus were evacuated. Histopathological examination was also performed. The wound was irrigated with 2 L of 0.9% NaCl and hecting was performed layer by layer subsequently. Specimens were collected and sent for microbiological and pathological analyses. The result of the AFB examination was based on types of grading scale by the World Health Organization and the International Union against Tuberculosis and Lung Disease (WHO-IUATLD) was 2+. Tissue culture was found to be positive. Histopathological examination showed granulomatous inflammation, swollen connective tissues containing epithelioid tubercle nests with necrotization, and datia Langhans cells ( Figure 4). The results were consistent with TB. Furthermore, the anti-human immunodeficiency virus (HIV) test was negative. The patient was subsequently diagnosed with TB of the elbow joint.

**Figure 5.**
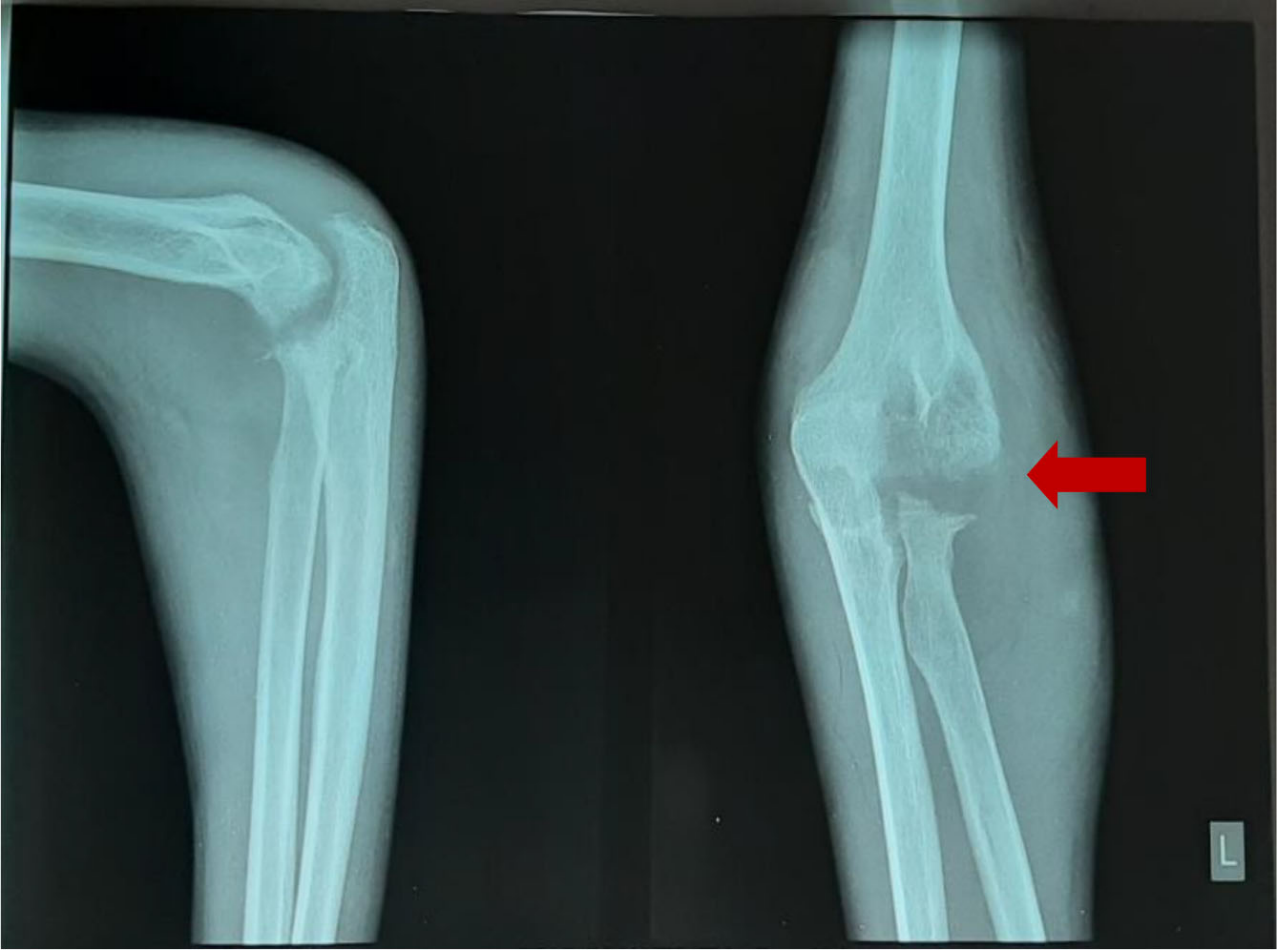
Minimal improvement, AP and lateral projection of the left elbow joint radiographs showed erosion of the distal cortex of the humerus and radial bone, destruction of the distal cortex, and swelling of the soft tissue of the left elbow area (March 2021).

**Figure 6.**
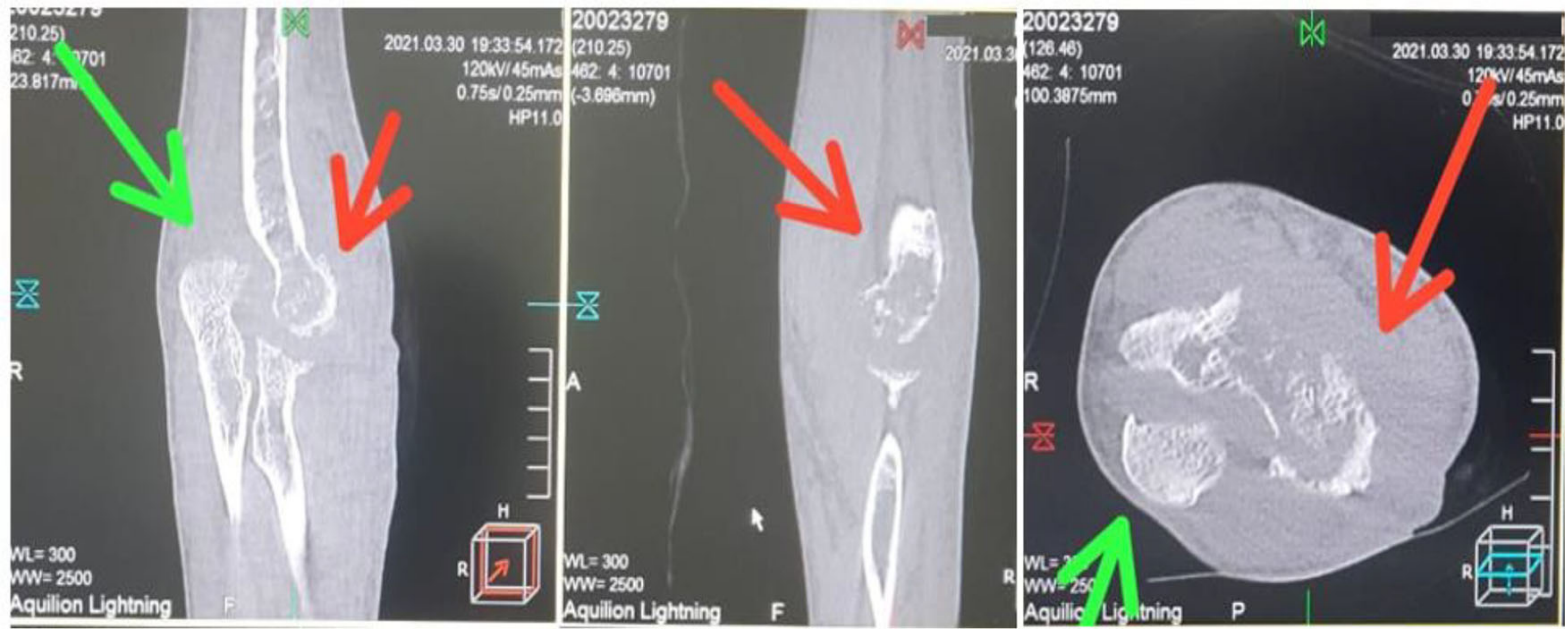
Computed tomography scan shows destruction of the lateral epicondylus of the humeral bone and the processus olecranon of ulna bones (red arrows = humerus bones, green arrows = ulna bones). Picture was edited with photoshop CS4 version 11.0 to remove specific details of dates of patient care and patient’s identity.

The patient was given a standard first-line oral regimen of extrapulmonary TB treatment; an intensive phase for two months with rifampicin 450 mg once daily, isoniazid 300 mg once daily, pyrazinamide 1000 mg once daily, and ethambutol 1000 mg once daily (2HRZE) and seven months of a continuation phase with rifampicin 450 mg once daily and isoniazid 300 mg once daily (7HR). The patient had been undergoing continuation phase of the treatment and his condition showing improvements, including decreased pain, increased appetite, and weight gain. However, flexion and extension are restricted. The patient reported clinical improvement and discharge was decreased. Left elbow joint radiographs showed minimal improvement ( Figure 5). Computed tomography (CT) scan results showed destruction of the lateral *epicondylus* of the humeral bone and the *processus olecranon* of ulna bones, after two months of the treatment ( Figure 6).

## Discussion

Musculoskeletal TB occurs in about 10% of all cases of extrapulmonary tuberculosis, which commonly affects weight-bearing joints such as spine (51%), pelvis (12%), hip and femur (10%), knee and tibia (10%). Reported cases of non-weight-bearing joints such as elbow joint TB are still relatively few, and the diagnosis still often to be neglected. ^1^

Reactivation of bacilli embedded in bone during the first mycobacteremia of primary infection causes tuberculous elbow and arthritis. The extensive vascular supply of the vertebra and growth plates of the long bones explains the bacillus' preference for the spine and major joints. Musculoskeletal tuberculosis develops as a result of the bacilli being seeded in the bloodstream shortly after the initial pulmonary infection. Osteoarticular tuberculosis begins as osteomyelitis in the growth plates of bones, where the blood supply is strongest, and subsequently spreads locally into joint spaces. It can also spread through the lymphatic system; however this is a less usual occurrence. The stimulation of dormant lymphatic or blood stream areas of morbidity might cause infections in joints. In the long bones, tuberculosis begins in the epiphysis and progresses to the marrow, where it causes tubercle formation and trabeculae infection. The mycobacteria cause an inflammatory response in the synovium of the joint, which is followed by the production of granulation tissue. The granulation tissue pannus then starts to erode and degrade cartilage and finally bone, resulting in demineralization. ^5^^–^^7^

Diagnosis of musculoskeletal TB requires the clinician’s ability to pay attention to joint swelling and chronic pain, as well as their effects on joint function. ^5^

Usually, respiratory and systemic symptoms are absent or only briefly present. In this report, only a history of fever was identified. Radiological examination of the lungs showed no abnormality. The complaints for joint TB are often non-specific, hence a late diagnosis. ^1^

The findings in this report are consistent with several previous studies. A study by Yazici *et al.* (2016) reported a TB of the elbow joint case in which there were no signs and symptoms of respiration. The results of chest radiographs were still within normal limits. The diagnosis was confirmed by AFB and histopathology examinations. ^8^ Another study by Guan & Zeng (2021) reported osteoarticular TB with a picture of swelling and pain that was previously diagnosed as osteoarthritis. Although these cases are rare, they are difficult to diagnose and can cause pain and impaired function. ^9^

Radiographic changes of the joints may suggest multiple osteolytic lesions and there may be erosions of the joints and swelling of the soft tissues. ^10^ Unfortunately, this patient did not undergo a magnetic resonance imaging examination due to the limited available facilities. Definite diagnosis required synovial fluid aspiration. Microscopic examination and culture of fluid aspiration were very helpful, followed by histopathological results showing the caseous granuloma. ^11^ These non-specific sign and symptoms were often delay the diagnosed as skeletal TB, as reported in one study that shows the time lag from the onset of complaints until the diagnosis was confirmed as approximately 4-11 months. Additionally, some cases of skeletal TB occasionally showed negative results on AFB and culture. ^12^^,^^13^

Clinicians should not neglect to explore the history of exposure and factors that increase the risks of TB infection such as close contact with confirmed TB patients, immunocompromise (e.g. HIV infection), diabetes *mellitus*, and having comorbid diseases such as chronic kidney disease. Therefore, it is necessary to screen the patient for co-infectious diseases listed above. Other risk factors are old age, poor nutrition, and receiving immunosuppressive treatments. ^6^ Regarding this case, the risk factors are not clear.

In summary, the significance of this case is the recognition of risk factors for skeletal TB and chronic symptoms, so that they can be treated properly. Early diagnosis and treatment can be achieved through with careful anamnesis that does not ignore the history of close contact with confirmed case TB patients, risk factors for TB infection, physical/clinical, radiological, and laboratory examinations. It is important for clinicians, especially those who work in an area endemic to suspect chronic joint pain whose clinical symptoms do not improve with conventional treatment as skeletal TB as the differential diagnosis. The specific AFB smear and culture tests are still important, although can occasionally show false negative results. Extrapulmonary TB can be deceptive because it does not always cause typical symptoms and pulmonary involvement. Prompt diagnosis and treatment are essential to prevent joint damage and impaired function.

## Patient’s perspectives

Left-arm and elbow pain, swelling, and immobility made me suffer. I knew that I had to go to the hospital for further treatment. However, I was afraid of surgery and at the suggestion of my family, I underwent traditional medicine with massage therapy for almost 1 year. I didn't expect that my illness would get worse and I had to have surgery immediately and take long-term medication. Now I feel better, my arm pain and swelling of my left elbow have decreased, even though I haven't been able to move my arm to its full potential.

## Data availability

All data underlying the results are available as part of the article and no additional source data are required.

## Consent

Written informed consent for publication of their clinical details and clinical images was obtained from the patient.
