# Supplementary material for: Case Report: Delayed treatment of tuberculosis of the elbow joint
Source: F1000Res. 2022 May 13;10:486. Originally published 2021 Jun 18. [Version 2] doi: 10.12688/f1000research.53488.2 (PMC9201408; doi:10.12688/f1000research.53488.2)
Supplement: Peer review history

# Peer review: Reviewer response for version 2

**DOI:** 10.5256/f1000research.133791.r137727

**Published:** 2022-06-15

**Authors:** Yunita Arliny

**Affiliations:** 1 Department of Pulmonology and Respiratory Medicine, Faculty of Medicine, Universitas Syiah Kuala, Banda Aceh, Indonesia

Changes made in version 2 are good and appropriate.

Are enough details provided of any physical examination and diagnostic tests, treatment given and outcomes?

Partly

Is the case presented with sufficient detail to be useful for other practitioners?

Yes

Is sufficient discussion included of the importance of the findings and their relevance to future understanding of disease processes, diagnosis or treatment?

Partly

Is the background of the case’s history and progression described in sufficient detail?

Partly

Reviewer Expertise:

pulmonary infection

I confirm that I have read this submission and believe that I have an appropriate level of expertise to confirm that it is of an acceptable scientific standard.

# Peer review: Reviewer response for version 2

**DOI:** 10.5256/f1000research.133791.r137728

**Published:** 2022-06-06

**Authors:** Musofa Rusli

**Affiliations:** 1 Department of Internal Medicine, Faculty of Medicine, Airlangga University, Surbaya, Indonesia

I have no further comments to make.

Are enough details provided of any physical examination and diagnostic tests, treatment given and outcomes?

Yes

Is the case presented with sufficient detail to be useful for other practitioners?

Yes

Is sufficient discussion included of the importance of the findings and their relevance to future understanding of disease processes, diagnosis or treatment?

Yes

Is the background of the case’s history and progression described in sufficient detail?

Yes

Reviewer Expertise:

Infectious disease

I confirm that I have read this submission and believe that I have an appropriate level of expertise to confirm that it is of an acceptable scientific standard.

# Peer review: Reviewer response for version 1

**DOI:** 10.5256/f1000research.56876.r127931

**Published:** 2022-04-04

**Authors:** Yunita Arliny

**Affiliations:** 1 Department of Pulmonology and Respiratory Medicine, Faculty of Medicine, Universitas Syiah Kuala, Banda Aceh, Indonesia

1. Reflexology that is done for complaints of elbow pain or pain in other places? Because it is somewhat contrary to the statement of elbow pain and does not seem to be related to delayed therapy.
2. On photo session, it's better if you include a photo of the elbow before the operation.
3. The sentence "The patient was subsequently diagnosed with TB of the elbow joint" should be placed after the results of AFB and histopathological examinations are obtained. the sentence "diagnosed with TB of the elbow joint" should be replaced with suspected TB elbow joint.
4. Please complete the result of AFB examination (IULTD scale) and culture.
5. Has the patients examined of Gene Xpert MTB/RIF from the tissue? Please mention this. 
6. Please add an explanation of the epidemiology of bone and joint TB (age, gender) and its pathogenesis.
7. Please explain the sentence "The specific AFB smear and culture tests are still important, although they can occasionally show false negative results.
8. Please mention the gold standard of diagnostics for bones or joint TB.

Are enough details provided of any physical examination and diagnostic tests, treatment given and outcomes?

Partly

Is the case presented with sufficient detail to be useful for other practitioners?

Yes

Is sufficient discussion included of the importance of the findings and their relevance to future understanding of disease processes, diagnosis or treatment?

Partly

Is the background of the case’s history and progression described in sufficient detail?

Partly

Reviewer Expertise:

pulmonary infection

I confirm that I have read this submission and believe that I have an appropriate level of expertise to confirm that it is of an acceptable scientific standard, however I have significant reservations, as outlined above.

# Response

**Published:** 2022-05-05

**Authors:** Desdiani Desdiani

**Affiliations:** Universitas Sultan Ageng Tirtayasa, Indonesia

**Dear Dr. Yunita Arliny,**

 Thank you for allowing us the opportunity to submit a revised draft of the manuscript “Case Report: Delayed treatment of tuberculosis of the elbow joint” for publication in the F1000Research.

 We appreciate the time and effort that you dedicated to providing feedback on our manuscript and are grateful for the insightful comments and valuable improvements to our paper. We have incorporated most of the suggestions made by the reviewers. Please see below, for a point-by-point response to the reviewers’ comments and concerns:

**Reflexology that is done for complaints of elbow pain or pain in other places? Because it is somewhat contrary to the statement of elbow pain and does not seem to be related to delayed therapy.**

 This patient complained about pain in his left elbow and noticed swelling. Initially, he complained about pain all over his left arm, after several reflexology massages to alleviate his toothache for almost 2 months. However, instead of seeking medical treatment, he visited a traditional massage therapist every week without improvement in his left arm pain including his left elbow for almost one year. At the first hospital visit, the elbow pain had gotten more severe and it became swollen. Discharge emerged from a small skin perforation located on the inner side of the left elbow. The patient finally went to the orthopedic department and underwent surgery.

**On photo session, it's better if you include a photo of the elbow before the operation.** Initially, this patient came to the orthopedic clinic and was treated because of severe pain and swelling in the elbow of the left arm. Unfortunately, the orthopedic specialist did not think to take a photo, he was immediately treated and underwent laboratory and radiological examinations, and then surgery was performed.

**The sentence "The patient was subsequently diagnosed with TB of the elbow joint" should be placed after the results of AFB and histopathological examinations are obtained. the sentence "diagnosed with TB of the elbow joint" should be replaced with suspected TB elbow joint.**

 We agree and have updated.

**Please complete the result of AFB examination (IULTD scale) and culture** .                 

 The result of the AFB examination is based on types of grading scale by the World Health Organization and the International Union against Tuberculosis and Lung Disease (WHO-IUATLD): **1–10 AFB per field, 2+** . Tissue culture was found to be positive.

**Has the patients examined of Gene Xpert MTB/RIF from the tissue? Please mention this.**

 Initially, this patient came to the orthopedic clinic and was treated because of severe pain and swelling in the elbow of the left arm. Unfortunately, the orthopedic specialist did not think to take a photo, he was immediately treated and underwent laboratory and radiological examinations, and then surgery was performed. The source of the sample for histopathological examination was taken from white granulation tissue on the posterior region of cubiti sinistra. Histopathological examination showed granulomatous inflammation, swollen connective tissues containing epithelioid tubercle nests with necrotization, and datia Langhans cells.

**Please add an explanation of the epidemiology of bone and joint TB (age, gender) and its pathogenesis.**

 We agree and have updated.

 In Europe and the United States, bone and joint TB accounts for 2.2–4.7 percent of all TBcases and roughly 10–15 percent of EPTB cases, whereas in developing countries, particularly Asia, the incidence of EPTB rises to 15–20 percent. Males and females have identical rates of infection with Mycobacterium tuberculosis until adolescence, following which males have a greater rate. For all ages, male rates became higher than female rates. Many developing countries' current notification rates for both sexes are comparable to those of industrialized countries in the mid-twentieth century, though the sex and age pattern is similar to that of industrialized countries today, with men's disease rates exceeding women's after the age of fifteen. These data raise the potential that tuberculosis cases among women in underdeveloped countries are underreported. The average age of tuberculous vertebral osteomyelitis patients is 45–60 years old. Nonetheless, some research show a bimodal age distribution, with two peaks, one between 20 and 30 years old, linked to immigration and/or HIV infection (60 percent of cases in one study), and the other between 60 and 70 years old. Simultaneous extraspinal involvement is seen in 5–50% of cases, while concomitant lung illness is seen in 2.3–65% of cases. The development of symptoms in spinal TB is usually gradual, and the disease progresses slowly, albeit an early onset has been documented. Symptoms might last anywhere from two weeks to many years at the time of diagnosis. The typical symptom duration was at least 12 months in early studies, although more current articles report a symptom duration of 2–7 months. Pain is common (83–100%), although only a third of individuals have a fever or other constitutional symptoms. Patients with extraspinal TB and those with disseminated disease are more likely to experience these symptoms. In tuberculosis of the upper limb, the elbow joint is the most commonly implicated joint. The incidence of elbow tuberculosis has been observed to range from 2 to 5% of all skeletal sites. There are only a few important reports on TB of the elbow joint that have been published. There were no management options or classifications. Histopathology, AFB staining, and polymerase chain reaction (PCR) were all performed on the samples.

**Pathogenesis**

 Reactivation of bacilli embedded in bone during the first mycobacteremia of primary infection causes tuberculous elbow and arthritis. The extensive vascular supply of the vertebra and growth plates of the long bones explains the bacillus' preference for the spine and major joints. Musculoskeletal tuberculosis develops as a result of the bacilli being seeded in the bloodstream shortly after the initial pulmonary infection. Osteoarticular tuberculosis begins as osteomyelitis in the growth plates of bones, where the blood supply is strongest, and subsequently spreads locally into joint spaces. It can also spread through the lymphatic system; however this is a less usual occurrence. The stimulation of dormant lymphatic or blood stream areas of morbidity might cause infections in joints. In the long bones, tuberculosis begins in the epiphysis and progresses to the marrow, where it causes tubercle formation and trabeculae infection. The mycobacteria cause an inflammatory response in the synovium of the joint, which is followed by the production of granulation tissue. The granulation tissue pannus then starts to erode and degrade cartilage and finally bone, resulting in demineralization. Proteolytic enzymes that damage peripheral cartilage aren't created because tuberculosis isn't a pyogenic infection. As a result, for a long period, the joint space is kept. Abscesses in the surrounding tissue may occur if the infection is allowed to proceed without treatment. Sequestration of bone is uncommon due to the absence of space-occupying exudates with substantial interruption of vascular supply. As a result, the active phase of tuberculous elbow is characterized by bone loss without sequestra and little new bone growth.

**Please explain the sentence "The specific AFB smear and culture tests are still important, although they can occasionally show false negative results.**

 A negative AFB smear indicates that there is no infection, that symptoms are caused by anything other than mycobacteria, or that mycobacteria were not present in sufficient numbers to be detected under a microscope. In order to increase the likelihood of finding the organisms, at least three samples are usually taken. However, if AFB smears are negative but a strong suspicion of a mycobacterial infection remains, more samples may be taken and analyzed on different days. Because the culture media permits tiny amounts of germs to thrive and be detected, a smear negative sample can nonetheless grow mycobacteria. AFB smears that are positive suggest a mycobacterial infection. To confirm a diagnosis, however, a culture must be taken. AFB smear results are combined with results from the nucleic acid amplification test (NAAT) for TB in persons who have signs and symptoms of an active TB infection. Though a culture is required for a conclusive diagnosis, the results of the smear and NAAT may be useful in determining what to do. Histopathology, AFB staining, and polymerase chain reaction all validated the diagnosis of elbow tuberculosis (PCR). Biopsy without affecting the bone's integrity. The histopathologic results such as epitheloid infiltration, tubercle formation, caseous necrosis, and Langhan's gaint cells confirmed the TB pattern.

**Please mention the gold standard of diagnostics for bones or joint TB.** Bone biopsy is the gold standard for diagnosis.

**References :**

 [1] Holmes CB, Hausler H, Nunn P. A review of sex differences in the epidemiology of tuberculosis. *Int J Tuberc Lung Dis* . 1998;2(2):96–104.

 [2] Dhillon M, Goel A, Prabhakar S, Aggarwal S, Bachhal V. Tuberculosis of the elbow: A clinicoradiological analysis. *Indian J Orthop.* 2012;46(2):200. DOI: https://doi.org/10.4103/0019-5413.93684

 [3] Haider Abdul-Lateef Mousa. Bones and Joints Tuberculosis. *Bahrain Med Bull* . 2007;29(1):17–21.

 [4] Pigrau-Serrallach C, Rodríguez-Pardo D. Bone and joint tuberculosis. *Eur Spine J.* 2013;22 Suppl 4(Suppl 4). DOI: https://doi.org/10.1007/S00586-012-2331-Y

# Peer review: Reviewer response for version 1

**DOI:** 10.5256/f1000research.56876.r88014

**Published:** 2021-07-05

**Authors:** Musofa Rusli

**Affiliations:** 1 Department of Internal Medicine, Faculty of Medicine, Airlangga University, Surbaya, Indonesia

The background and risk factors of the case and the case’s progression are sufficiently described. In addition, there are enough details provided of the physical examination, diagnostic tests, and brief outcome of the case. Furthermore, a good discussion is included of the importance of the findings and their relevance in managing the particular case. Finally, there is an adequate presentation of the clinical management to be useful for other practitioners.

 However, some issues need to be taken into account. For example, it is better to use a proofreading service to correct some typographical mistakes. In the Case Report Section, the source of sample in the sentence “histopathological examination was also performed” needs to be clarified; is it from the pus or other tissue?

 Below are some references worth considering to be included in the manuscript:

- Boodoo *et al.* (2020 ^1^)
- Liao *et al. *(2017 ^2^)

Are enough details provided of any physical examination and diagnostic tests, treatment given and outcomes?

Yes

Is the case presented with sufficient detail to be useful for other practitioners?

Yes

Is sufficient discussion included of the importance of the findings and their relevance to future understanding of disease processes, diagnosis or treatment?

Yes

Is the background of the case’s history and progression described in sufficient detail?

Yes

Reviewer Expertise:

Infectious disease

I confirm that I have read this submission and believe that I have an appropriate level of expertise to confirm that it is of an acceptable scientific standard.

## References

[1] : Osteoarticular tuberculosis involving the elbow. *Oxf Med Case Reports* .2020;2020(10) : 10.1093/omcr/omaa085 omaa085 10.1093/omcr/omaa085 33133618PMC7583421

[2] : Mycobacterium tuberculosis of the elbow joint. *BMJ Case Rep* .2017;2017: 10.1136/bcr-2017-222735 10.1136/bcr-2017-222735 29217612PMC5728216

# Response

**Published:** 2021-07-13

**Authors:** Desdiani Desdiani

**Affiliations:** Universitas Sultan Ageng Tirtayasa, Indonesia

Dear Dr. Musofa Rusli,

 Thank you for allowing us the opportunity to submit a revised draft of the manuscript “Case Report: Delayed treatment of tuberculosis of the elbow joint” for publication in the F1000Research.

 We appreciate the time and effort that you dedicated to providing feedback on our manuscript and are grateful for the insightful comments and valuable improvements to our paper. We have incorporated most of the suggestions made by the reviewers. Please see below, for a point-by-point response to the reviewers’ comments and concerns:

1. We have used a proofreading service to correct some typographical mistakes with scribendi.com
2. The source of the sample for histopathological examination was taken from white granulation tissue on the posterior region of *cubiti sinistra*.
3. We are agree that references such as Voodoo et al. (2020) and Liao et al. (2017) can be used for comparison of clinical, histopathological and radiological findings with the patients we analyzed and reported at this time.

Thank you so much, 

 Best Regards,

 Desdiani Desdiani

# Response

**Published:** 2022-06-15

**Authors:** Desdiani Desdiani

**Affiliations:** Universitas Sultan Ageng Tirtayasa, Indonesia

Dear Musofa Rusli,

 Thank you for your respond and review.

 Best Regards,

 Desdiani Desdiani
